# Supplementary material for: Tracking Progress from Policy Development to Implementation: A Case Study on Adoption of Mandatory Regulation for Nutrition Labelling in Malaysia
Source: Nutrients. 2021 Jan 29;13(2):457. doi: 10.3390/nu13020457 (PMC7910920; doi:10.3390/nu13020457)
Supplement: Supplementary file 1 [file nutrients-13-00457-s001.zip › 5. Supplementary Material 3 Historical Mapping (Label).pdf]

Supplementary Material 3 Full Historical Mapping of Mandatory Nutrition Labelling Case in Malaysia

The global initiation of a nutrition labelling policy agenda began through the *Codex Committee on Food Labelling* in 1985, with the Guidelines on Nutrition Labelling focussing on energy, protein, available carbohydrate and fat for mandatory nutrition labelling, along with considering its application to any nutrient claim [1]. However, participants did not signify there was any response at the local level at this time. Instead, the rising trends in Malaysia of over nutrition and NCDs in the 1980's to 1990's [2-4] triggered a series of government-led prevention actions, which evolved through *the Healthy Lifestyle Awareness* campaigns 1991-2002. From these activities, 'reading food labels' emerged as part of the health promotion approach to promoting a healthy diet [5-6].

In 1992, the *World Declaration on Nutrition* mooted at the first *International Conference on Nutrition* by the FAO and WHO [7], lead to a global *Plan of Action for Nutrition* for governments. The global plan recommended harmonisation of food labelling with Codex standards for consumer protection, and the use of nutrition labelling to promote healthy diets and lifestyles through appropriate community-based nutrition education programmes [7]. Participants highlighted that this catalysed action in Malaysia, resulting in the initiation of the National Plan of Action for Nutrition of Malaysia (NPANM) 1996-2000 to align with the global plan.

Global political support for the *Codex Alimentarius* was fostered through the establishment of the World Trade Organization (WTO) in 1995 [8]. For example, enforcement of the *Sanitary and Phytosanitary* and *Technical Barriers to Trade*

agreements referred to the Codex guidelines. In Malaysia, the mandatory nutrition labelling at that time only applied to foods such as infant formula, cereal-based foods for infants and young children [9]. A recommendation to expand further requirements for nutrition labelling alignment to the *Codex Alimentarius* standards was initiated under the NPANM 1996-2000 [3]. This timepoint (1996) coincided with the appointment of Food Safety and Quality Division, Ministry of Health Malaysia as the Codex Contact Point [10].

The government initiated a proposal for food regulations around the year 2000, seeking public comments and conducting a series of seminars such as the 'National Seminar on Nutrition Labelling: Regulations and Educations' [9,11-13]. The mandatory nutrition labelling (P.U. (A) 88, Reg. 18B) was gazetted on 31st March 2003 with a few extensions in the enforcement date until 30th June 2005 [14-15]. In 2005, the *National Nutrition Policy of Malaysia* was introduced with a strategy focusing on public accessibility to nutrition information and education, as well as resources and opportunities to make informed decisions on healthy food choices [16]. In the same year, the *Guide to Nutrition Labelling and Claims* was published and regularly updated [17-19] to facilitate policy implementation in Malaysia.

At the international level, the *Global Strategy on Diet, Physical Activity and Health* (DPAS) was endorsed at the 57th WHA [20]. Subsequently, the *Codex Alimentarius Commission* agreed to the DPAS implementation in 2005 [21], triggering the revision of the Codex guidelines on the list of mandatory nutrient declarations [22]. In Malaysia, interest in aligning to the Codex agenda was apparent, as a review of food regulations for the list of mandatory foods and nutrients was planned under the NPANM II 2006-

2015 [23]. However, there was some reservation on adopting nutrient declarations pertaining to *trans* fatty acids (TFA). The proposal of mandatory TFA labelling was mooted in 2008 by the Malaysian government but because local food product market surveys revealed low TFA content, no further action was taken [24]. Two events in 2009 were the mandatory nutrition labelling for instant noodles [24] and the gazetting of an explicit definition of 'total sugars' and the format of fatty acids declaration [25]. In particular the mandatory labelling related to instant noodles was mooted by the Cabinet.

The *Codex Alimentarius Commission* expanded the list of mandatory nutrient declarations to include total sugars, sodium and saturated fat in 2011 [26]. Although participants did not cite significant national activities during the period 2011 and 2015, some external events were cited as important by relevant stakeholders in Malaysia. Through the *Rome Declaration on Nutrition* in 2014, governments committed to creating an enabling environment for consumers to make informed food choices through nutrition information and education [27]. In the same year, *Consumers International* called upon the *World Health Assembly* to establish a global convention for healthy diet for populations [28]. Together with *World Obesity* they called for nutrition declarations of countries to be in line with the Codex principles [29]. *Consumers International* later launched the 'healthy diet' theme for the *World Consumer Rights Day* in 2015 [28].

Under the NPANM III 2016-2025, the Malaysian government set future plans to introduce mandatory sodium and total sugars declarations for all food products and declaration of four types of fatty acids for salad dressing, flour confectionery, milk and

cereal categories [30]. While these plans were in gestation, the introduction of a voluntary *Healthier Choice Logo* (HCL) in 2017 provided an indirect mandate. Products carrying HCL endorsement for any specific nutrient relating to sodium, total sugars, saturated fat and/or trans-fat content were required to display the relevant nutrient declaration [31-32].

References:

1. Joint FAO/WHO Codex Alimentarius Commission. *Report of the Seventeenth Session of the Codex Committee on Food Labelling Ottawa, Canada, 12 – 21 October 1983 (ALINORM 85/22)*. WHO & FAO: Rome, 1985.
2. Khor, G.L.; Gan, C.Y. Trends and dietary implications of some chronic non-communicable diseases in peninsular Malaysia. *Asia Pac J Clin Nutr.* **1992**, *1* (3), 159-168.
3. National Coordinating Committee on Food and Nutrition (NCCFN). *National Plan of Action for Nutrition of Malaysia (1996-2000)*. Ministry of Health, NCCFN: Kuala Lumpur, 1995.
4. Noor, M.I. The nutrition and health transition in Malaysia. *Public Health Nutr.* **2002**, *5* (1a), 191-195.
5. Bahagian Pendidikan Kesihatan. Kempen Cara Hidup Sihat 1997. 2017. Available online: https://www.infosihat.gov.my/index.php/kchs/tahun-1997 (accessed on 10 January 2020).
6. Ministry of Health (MOH). Panduan Penyediaan Makanan Sihat. 1997. Available online: https://www.infosihat.gov.my/images/media_sihat/kchs/KCHS_1997/pdf/pameran/POSTER_1997_PDF_19.pdf (accessed on 10 January 2020).
7. Food and Agriculture Organization of the United Nations & World Health Organization (FAO & WHO). International Conference on Nutrition – Final Report of the Conference (Rome, December 1992). 1992. Available online: https://apps.who.int/iris/bitstream/handle/10665/61254/a34812.pdf;jsessionid=B1

125DB78948B254FE9155A90855C036?sequence=1 (accessed on 18 March 2020).

8. Veggeland, F.; Borgen, S.O. Negotiating International Food Standards: The World Trade Organization’s Impact on the Codex Alimentarius Commission. *Governance* **2005**, *18* (4), 675-708.
9. Tee, E.S.; Tamin, S.; Ilyas, R.; Ramos, A.; Tan, W.L.; Lai, D.K.S.; et al. Current status of nutrition labelling and claims in the South-East Asian region: Are we in harmony? *Asia Pac J Clin Nutr.* **2002**, *11* (2), S80-S86.
10. Food Safety and Quality Division (FSQD). National Codex Committee (JKC). 2020. Available online: http://fsq.moh.gov.my/v6/xs/page.php?id=245 (accessed on 10 January 2020).
11. Food Safety and Quality Division (FSQD). *Proceedings National Seminar on Nutrition Labelling: Regulations and Education (7-8 August 2000)*. Ministry of Health, FSQD: Kuala Lumpur, 2000.
12. Ministry of Health (MOH). *Laporan Tahunan 2000: Kementerian Kesihatan Malaysia*. MOH: Putrajaya, 2000.
13. Ministry of Health (MOH). *Laporan Tahunan 2001: Kementerian Kesihatan Malaysia*. MOH: Putrajaya, 2001.
14. Food Safety and Quality Division (FSQD). *Annual Report 2003*. Ministry of Health, FSQD: Kuala Lumpur, 2003.
15. Ministry of Health (MOH). *Annual Report 2004: Ministry of Health Malaysia*. MOH: Putrajaya, 2004
16. Ministry of Health (MOH). *National Nutrition Policy of Malaysia*. MOH: Putrajaya, 2005.

17. Food Safety and Quality Division (FSQD). *Panduan Pelabelan Dan Akuan Pemakanan*. Ministry of Health, FSQD: Putrajaya, 2005.
18. Food Safety and Quality Division (FSQD). *Guide to Nutrition Labelling and Claims [as at December 2007]*. Ministry of Health, FSQD: Putrajaya, 2007.
19. Food Safety and Quality Division (FSQD). *Guide to Nutrition Labelling and Claims as at December 2010*; Ministry of Health, FSQD: Putrajaya, 2010.
20. World Health Organization (WHO). *Global Strategy on Diet, Physical Activity and Health*. WHO: Geneva, Switzerland, 2004.
21. Joint FAO/WHO Codex Alimentarius Commission. *Report of the Thirty-fourth Session of the Codex Committee on Food Labelling, Ottawa, Canada, 1-5 May 2003 (ALINORM 06/29/22)*. WHO & FAO: Rome, 2006.
22. Joint FAO/WHO Codex Alimentarius Commission. *Report of the Thirty-sixth Session of the Codex Committee on Food Labelling, Ottawa, Canada, 28 April - 2 May 2008 (ALINORM 08/31/22)*. WHO & FAO: Rome, 2008.
23. National Coordinating Committee on Food and Nutrition (NCCFN). *National Plan of Action for Nutrition of Malaysia (2006-2015)*. Ministry of Health, NCCFN: Putrajaya, 2006.
24. National Coordinating Committee on Food and Nutrition (NCCFN). *Mid-term review National Plan of Action for Nutrition of Malaysia (NPANM) 2006-2015*. Ministry of Health, NCCFN: Putrajaya, 2011.
25. Attorney-General's Chambers. Federal Government Gazette – Food (Amendment) (No.2) Regulations 2009 (P.U. (A) 306). 2009. Available online: http://www.lawnet.com.my/EGazette/Download?id=14363 (accessed on 5 February 2020).

26. Joint FAO/WHO Codex Alimentarius Commission. *Report of the Thirty-fourth Session of the Codex Alimentarius Commission, Geneva, Switzerland, 4-9 July 2011 (REP11/CAC)*. WHO & FAO: Rome, 2011.
27. Food and Agriculture Organization of the United Nations & World Health Organization (FAO & WHO). Second International Conference on Nutrition – Conference Outcome Document: Rome Declaration on Nutrition (Rome, 19-21 November 2014). 2014. Available online: http://www.fao.org/3/a-ml542e.pdf (accessed on 18 March 2020).
28. Consumers International. World Consumer Rights Day 2015 – Healthy Diets. n.d. Available online: https://www.consumersinternational.org/what-we-do/world-consumer-rights-day/wcrd-2015-healthy-diets (accessed on 11 January 2020).
29. Consumers International & World Obesity. Recommendations towards a Global Convention to protect and promote healthy diets. 2014. Available online: https://www.consumersinternational.org/media/2211/recommendations-for-a-convention-on-healthy-diets-low-res-for-web.pdf (accessed on 11 January 2020).
30. National Coordinating Committee on Food and Nutrition (NCCFN). *National Plan of Action for Nutrition of Malaysia (NPANM III) 2016–2025*. Ministry of Health, NCCFN: Putrajaya, 2016.
31. Nutrition Division. *Guidelines on Healthier Choice Logo Malaysia*. Ministry of Health, Nutrition Division: Putrajaya, 2017.
32. Nutrition Division. *Nutrient Criteria Healthier Choice (HCL) Malaysia*. Ministry of Health, Nutrition Division: Putrajaya, 2017.
