# Supplementary material for: Tracking Progress from Policy Development to Implementation: A Case Study on Adoption of Mandatory Regulation for Nutrition Labelling in Malaysia
Source: Nutrients. 2021 Jan 29;13(2):457. doi: 10.3390/nu13020457 (PMC7910920; doi:10.3390/nu13020457)
Supplement: Supplementary file 1 [file nutrients-13-00457-s001.zip › 3. Supplementary Material 1 Interview guide.pdf]

Supplementary Material 1 Interview guide

Subject Code	CS - 0__		
Name of Interviewer			
Date of Interview			
Start Time		End Time	
Duration (hr)			

Starting from now onward, I will address you (or title) with a generic term such as 'you, your, he, his' to protect your confidentiality. During this interview, the conversation will be audio-recorded and later transcribed into text for analysis. We assure you that all information that you provide will be stored securely and findings will be used for academic purpose (e.g. PhD thesis, scientific journals or presentation), and that you will not be identifiable in these materials.

1. My deep appreciation to you for participating in this research project and giving your precious time for the interview, which will be expected to take about 45 minutes to 1 hour and 30 minutes, depending on the issues discussed.

2. **Study aim:** To provide in-depth research insights and locally-grounded understanding of the process of food policy implementation and how different **barriers** and **facilitators** may affect the implementation. We are exploring this from different stakeholder perspectives (e.g. government, industry and civil society).

3. **Methods:** We are exploring two policy areas as case studies: (1) mandatory nutrition labelling; and/ or (2) restricting unhealthy food promotion in children's settings and broadcast media.

Before we begin the interview, it will be good to have your background information to facilitate the discussion. Please fill in the details below:

Sociodemographic Data of the Subject

**Age of the subject**	____________ years
**Gender**	◻ Male<br>◻ Female
**Ethnicity**	◻ Malay<br>◻ Chinese<br>◻ Indian<br>◻ Others: ___________
**Highest Education Level**	◻ No formal education<br>◻ Primary school<br>◻ Secondary school<br>◻ Diploma/ A-Level/ STPM<br>◻ Degree<br>◻ Master<br>◻ PhD
**Area of Expertise/ Working experience**	
**Years of experience in related field**	____________ years
**Identify your work area**	◻ Policy implementers/ public sector<br>◻ Food industry representatives/ private sector<br>◻ Non-government organisation<br>◻ Academia<br>◻ Professional: ____________________<br>◻ Others: ____________________
**Contact details (optional)**	◻ Yes<br>○ Phone number : __________________<br>○ Email : __________________<br>◻ No
**We will transcribe the conversation today into transcript. Do you wish to check?**	◻ Yes<br>◻ No

Screening Questions

Questions below will determine the level of discussions during the interview session. Please answer this session carefully. If you have any problem understanding the questions OR require further clarification, please ask the interviewer.

Q1 Do you have any experience of, or information related to, the policy development and/or implementation of <u>nutrition food labelling</u>?

- ☐ Yes ---- include **Session A**
- ☐ No

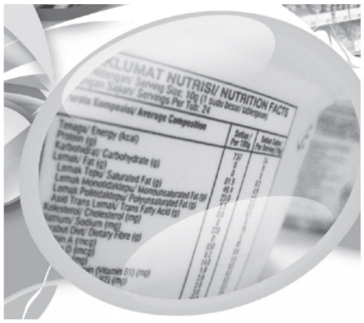

NUTRITION INFORMATION		
Serving size: 200 ml<br>Servings per package: 5		
	Per 100 ml	Per serving (200 ml)
Energy	100 kcal (420 kJ)	200 kcal (840 kJ)
Carbohydrate	23.8 g	47.6 g
Total sugars[1]	11.5 g	23.0
Protein	1.1 g	2.2 g
Fat	0 g	0 g

1 Declaration of total sugars is mandatory for ready-to-drink beverages only

Q2 Do you have any experience of, or information related to, policy development and/or implementation of <u>restricting unhealthy food promotion to children</u>?

- ☐ Yes ---- include **Session B**
- ☐ No

GARIS PANDUAN PENGIKLANAN DAN PELABELAN MAKLUMAT PEMAKANAN MAKANAN SEGERA

1. Pengenalan

Tujuan garis panduan ini adalah sebagai rujukan untuk indust[illegible] segera, agensi pengiklanan dan badan penyiaran dalam mengik[illegible] melabelkan maklumat pemakanan pada produk makanan segera. In[illegible] bagi garis panduan ini akan dimulakan pada tahun 2008. Bagi tujuan[illegible] pihak industri makanan segera hendaklah mengemukakan cadanga[illegible] ke Bahagian Keselamatan dan Kualiti Makanan untuk semakan dan k[illegible]

Malaysian Food and Beverage Industry's "Responsible Advertising to Children" Initiative
(The Malaysia Pledge)

Introduction:
The Malaysian pledge is an initiative to demon[illegible] of food and beverage to children. Signatories [illegible] Code of Advertising Practice.

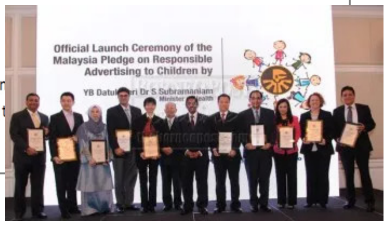

Thank you for answering the screening questions. Based on your answers, your critical insights into case studies for…...

- ☐ Nutrition food labelling (Session A)
- ☐ Restriction on unhealthy food promotion to children (Session B)

…… are crucial to us.

1. I would first describe to you a scenario setting using a diagram.
2. Next, I prepared some questions related to the scenario and would like to have your opinions.
3. Do you have any question so far?
4. If no, please sign the consent form (if not done) and I will check my recorder.

I will start the recording after showing you the diagram, and take notes throughout the conversation. Please do not hesitate to stop me, if you wish to.

Session A **(only if it is relevant to the interviewee based on the screening questions):**

Nutrition food labelling in Malaysia

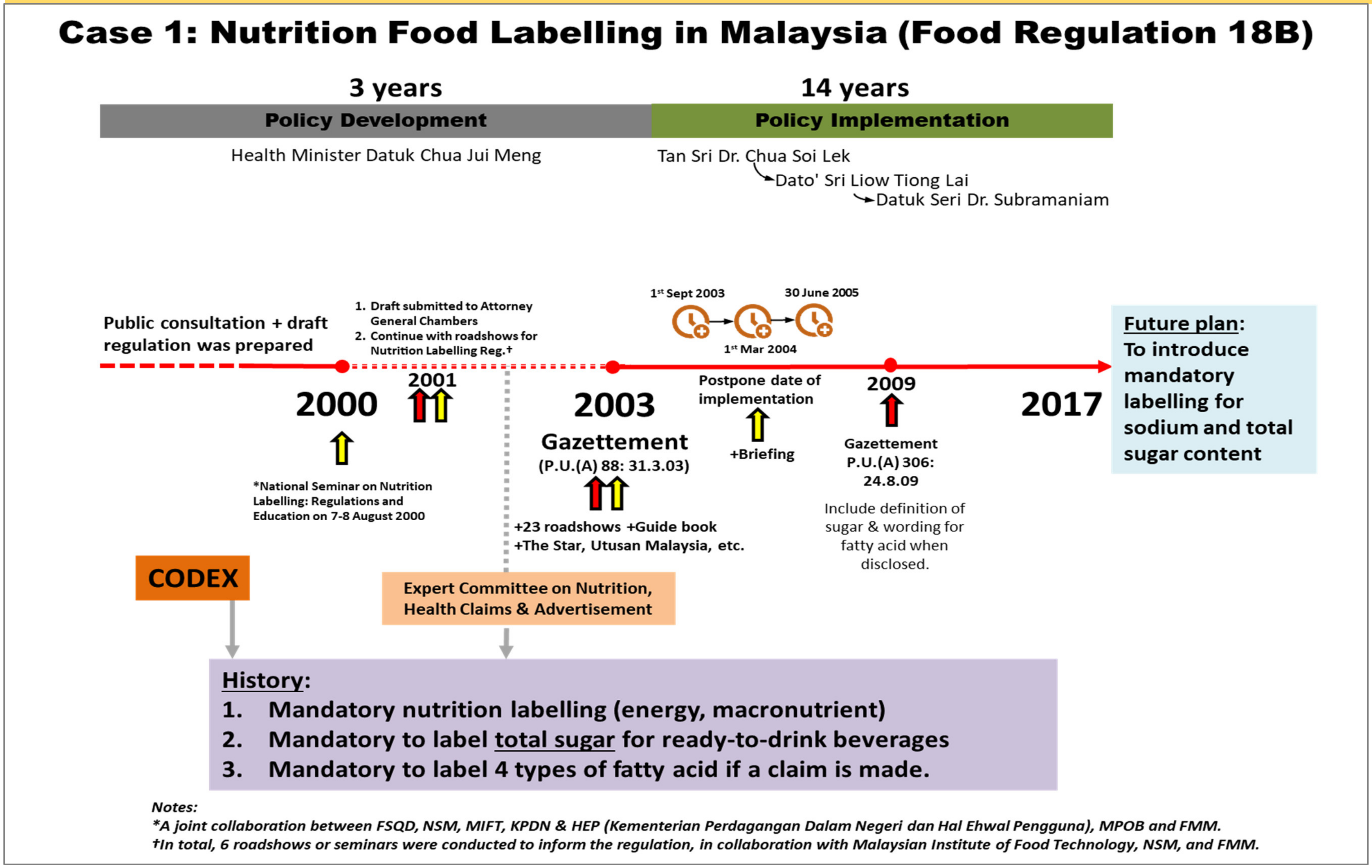

Figure 1 Nutrition food labelling in Malaysia

[Script read by the interviewer to explain Figure 1]:

1. We speculated the public consultation of the nutrition labelling regulations was conducted near 2000.
2. A national seminar on “Nutrition Labelling Regulations and Education” was conducted in 7-8 August 2000. This seminar was officiated by Minister of Health and invited speakers from USA, Secretariat Codex, Australia New Zealand Food Authority (ANZFA), Singapore and Thailand) and local speakers to present their proposals. About 380 participants attended the seminar.[1]
3. In 2001, a draft regulation was submitted to the Attorney General Chambers.[2]
4. The window of opportunities occurring between 2000 and 2003, led to policy development for nutrition food labelling. During this period or later, noticeable roadshows, seminars and/ or briefings were conducted.[3,4]
5. In March 2003, mandatory nutrition labelling was gazetted for energy, carbohydrate, protein and fat for 58 types of food products under Food Regulations 1985. Whereas, there are mandatory labelling for total sugar content of ready-to-drink beverages; and for 4 types of fatty acid content if a claim is made.
6. The Expert Committee on Nutrition, Health Claims & Advertisement instigated the development process together with other external and internal events.
7. Based on literature review, we identified that the implementation date was postponed a few times. First date was proposed on 1st Sept 2003, followed by 1st March 2004[5] and 30 June 2005[6].
8. In 2009, amendments (P.U.(A) 306 focused on the definition of sugars (all monosaccharides and disaccharides either added or naturally occurring) and format of declaration for fatty acid.
9. For your information, nutrition food labelling policies in Malaysia were rated by the experts as the highest score with medium implementation, against international best practice under the assessment of Food-Environment Policy Index (a benchmarking tool).
10. After almost 14 years since gazettement in 2003, as per the latest national nutrition plan, the government proposes to mandate nutrition labelling for sodium and total sugar content in all food product in stages on the food labels.

---

[1] MOH 2000, *Laporan Tahunan 2000: Kementerian Kesihatan Malaysia,* MOH.
[2] MOH 2001, *Laporan Tahunan 2001: Kementerian Kesihatan Malaysia,* MOH.
[3] MOH 2001, *Laporan Tahunan 2001: Kementerian Kesihatan Malaysia,* MOH.
[4] FSQD 2003, *Annual Report 2003,* Food Quality Control Division, MOH: Kuala Lumpur.
[5] FSQD 2003, *Annual Report 2003,* Food Quality Control Division, MOH: Kuala Lumpur.
[6] MOH 2004, *Annual Report 2004: Ministry of Health Malaysia,* MOH: Putrajaya.

[START RECORDING]

Key Questions:

1. Please tell us briefly about yourself.

   (*Prompts: current/ previous jobs, job's scope, professional roles or sector's role in policy*)

Session A: Semi-structured questions related to mandatory nutrition labelling

2. What were you doing at the time of the policy development and implementation?
   a. Can you tell me how you have been involved in the development and/or implementation of mandatory nutrition labelling?

      (Prompts – for secondary data: If you have not been directly involved, can you tell me what you know about the development and implementation of nutrition labelling)

3. **There are likely to be several factors that fostered the gazetting on nutrition labelling in 2003 (i.e. Planning and Development Phase).**

   a. What can you tell me about the process?
      (*Probe – was it smooth? or convoluted/ complex?*)

   b. Are you aware of factors that contributed to this?
      i. Who was involved?
         (Probe – Government, industry, NGOs and etc., how it was conducted. Note: Nutritionist, Dietitians, Food Scientists and Medical Doctors from various department and academicians involved as per FSQD feedback. The role of National Food Safety and Nutrition Council (NFSNC)?)
      ii. What were the key arguments and support for developing the policy at that time?
      iii. Any key event which might have precipitated/ led it to happen?
      iv. Can you relate if and how the media reported this issue?
         (Probe - What was the media coverage?)

c. **<u>Back to the policy itself</u>**, can you describe the scope of the policy when it was first proposed in the early development phase?

(Probe – Did it appear that the final policy was different from what was first proposed? Can you describe the factors / reasons why? What were the negotiations that went on to get to the final policy? According to CAC/ GL 2-1985 Codex Guidelines on Nutrition Labelling, it includes sodium and total sugar).

[Instruction: **Towards the end of the questions above, if the interviewee does not mention about the components and key prompts as identified based on literature reviews, the following questions can be used as a guide to probe more specific information.]**

d. [OPTIONAL AND EXAMPLE]

You have shared an insightful view on the process. In our scoping of this issue, we also identified a range of other possible aspects that may have influenced the development of mandatory nutrition labelling. Can you comment on the influence of (use prompts e.g. civil society) on the development of the policy?

(If the interviewee indicates the key prompts, then you can probe further. For example – How did Codex precipitate the development of the policy? Why? When did this occur?)

*Prompts (*these components can be vice-versa):*

**Components**	**Key Prompts (do not mention, unless the interviewee indicates)**	**Probe, if necessary (do not mention, unless the interviewee indicates)**
Government	Coalition/ Partnership	Who, what, when, why and how
Private Sector	Leadership	Who, when, why and how
Civil Society	Public consultation	When, what and how
Facilitators	Political will/ awareness	When, how and why
Barriers	Cost	What, why or how
	Resources	What, why and how
	Codex and trade	How, why and when
	International agenda	How, what and when

i. **Facilitators – leadership, positive response from public consultation.*
ii. *Government coalition – Ministry of Health and which other Ministries; established Expert Committee on Nutrition, Health Claims & Advertisement – involved nutritionists, dietitians, medical doctors, food scientists, academia.*
iii. *External factors e.g. media, advocacy from civil society, political events, economic conditions, influence from the Codex and trade, advocacy of international agenda such as the Global Strategy of Diet, Physical Activity and Health.*
iv. *Roles of private sector – forming coalition within industries (e.g. Federation of Malaysian Manufacturers), share common beliefs or vice-versa, applied resources e.g. media to advocate their agendas; corporate political activities highlighted by the food companies.*
v. **Barriers – low political awareness, incurred additional printing cost, lack of expertise and public advocacy.*

4. Now, I am moving to the period from 2004 until current, after gazetting of the nutrition labelling in 2003. I would also like you to think about any future plan of the government in relation to mandatory nutrition labelling.

a. Based on your experience or observation, what can you tell me about the implementation of this policy for the past 13 years?
(Probe – was it smooth? or full of obstacles? poorly, moderately or well implemented?)

b. Are you aware of factors that contributed to the implementation?

Prompts, if necessary:

i. Who was involved?
(Probe – Government, industry, NGOs and etc.)
ii. What were the key arguments and support for the implementation?
iii. Any key event which might precipitate the implementation?
iv. Did you observe any influence around policy content?
v. Could you recall if there was any discussion in the media?
(*Prompt also the future plan by the government)*

[Instruction: Towards the end of the questions above, if the interviewee does not mention about the components and key prompts as identified based on literature reviews, the following questions can be used as a guide to probe more specific information.]

c. [OPTIONAL AND EXAMPLE]

We have been discussing (recap what have been discussed). However, other people or literature indicated (use prompts e.g. the role of government), which you have not mentioned. Would you like to comment on this?

(If the interviewee indicates the key prompts, then you can probe further. For example – What is the implementation approach? Why is this approach selected? How does this impact?)

*Prompts (*these components can be vice-versa):*

**Components**	**Key Prompts (do not mention, unless the interviewee indicates)**	**Probe, if necessary (do not mention, unless the interviewee indicates)**
Government	Implementation approach	What, why, how
Private Sector	Organisation structure	What, who and how
Civil Society	Political will, perception and commitment	What and how
Facilitators	Resources	What, when and how
Barriers	A demand for change	What and why
	Internal and external factors	What, why and how

i. **Facilitators – proper collaborative approach; prioritised by the organisation (e.g. key performance index), structured framework (e.g. guideline, support) and trained staffs, external factors.*

ii. **Barriers – lack of individualised approach (e.g. top-down) and engagement with other departments, implementer's perception, organisation norm, high cost or profit margin reduction (e.g. loss of profit and employment, affect supply and demand paradigm), nature of organisation, difficult to integrate (e.g. vary format or parties involved which led to challenges and time consuming), low consumer demand, lack of evidence to support the formulation, difficulty in interpretation, lack of infrastructure* (e.g. human resources, no specialisation), existing legal or policy conflicts (e.g. self-regulation is stated in regulation), corporate political activities.*

5. If you could change the policy, what would you want to suggest?

Prompts: Who should do what and how? How do you think your ideas will make a difference to improve this policy?

Thanks for your input so far. We are about [half way] through. In the next part, we will discuss on policies related to ……

Session B (only if it is relevant): Restriction on unhealthy food promotion in children's settings and broadcast media

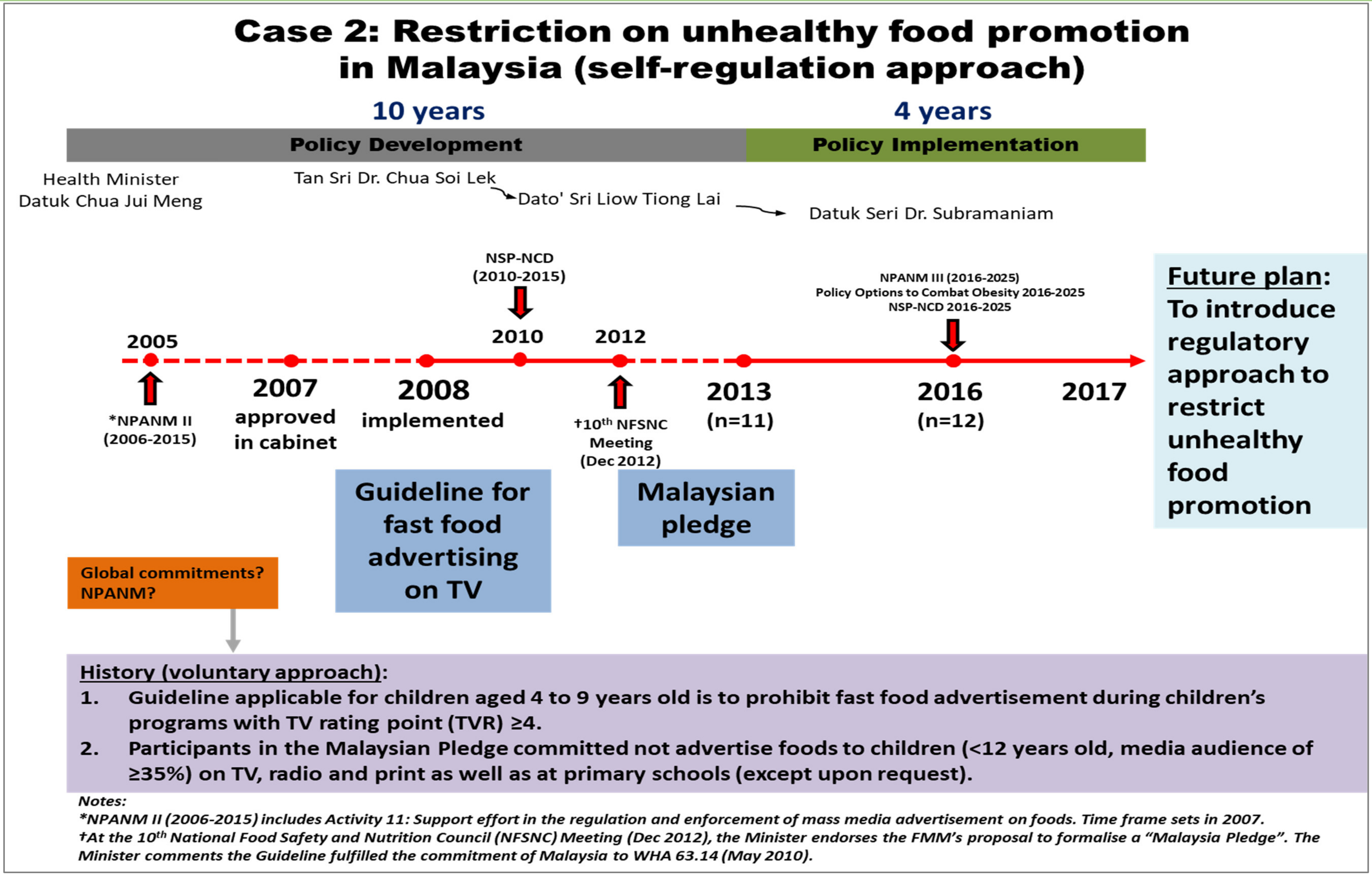

Figure 2 Restriction on unhealthy food promotion in Malaysia

[Script read by the interviewer to explain Figure 2]:

1. According to the National Plan of Action for Nutrition of Malaysia II (2006-2015) launched in 2005, there was an 'Activity 11' related to "regulation and enforcement of mass media advertisements on foods" with a time frame set in 2007.
2. Later, a Guideline was approved in the cabinet in 2007. It was implemented in 2008 as a self-regulation approach to prohibit fast food advertisement to target on children's programs (i.e. for children aged 4 to 9 years old at children's programs with TV rating ≥4).
3. In 2010, according to the National Strategic Plan for Non-communicable Diseases (NSP-NCD), it identified the role of Ministry of Information, Communication, Arts and Culture as to introduce "regulation of advertisements on unhealthy food/ drinks to children".
4. In 2012, at the 10th National Food Safety and Nutrition Council (NFSNC) Meeting (13 Dec 2012), the Health Minister endorses Federation of Malaysian Manufacturers' (FMM) proposal to formalise a "Malaysia Pledge - Responsible Advertising to Children". The Minister comments that the Guideline fulfilled the commitment of Malaysia to WHA 63.14 (May 2010).
5. In 2013, the Malaysian Food & Beverage Industry's Responsible Advertising to Children Initiative was introduced with 11 participating food companies (increased to 12 in 2016) to commit not to advertise foods to children (<12 years old, media audience of ≥35%) on TV, radio and print as well as at primary schools (except upon request).
6. According to the latest national nutrition plans (e.g. NPANM III, Policy Options to Combat Obesity, NSP-NCD II, etc.), there is an intention by the government to introduce a regulatory approach to control unhealthy TV food promotion to children, by 2020).
7. For your information, under the assessment of Food-Environment Policy Index (a benchmarking tool), the experts rated these policies as the lowest scores with low implementation, against international best practice.
8. Critical time points related to policy development for the Guideline occurred before 2007/2008. Whereas for the Malaysian Pledge, it occurred before 2012/2013.

Remarks:

a. *WHA 57.17 (2004) Global Strategy on Diet, Physical Activity & Health 2004 called on industry to practice responsible marketing of foods high in SFA, TFA, added sugars and salt (especially to children).*

b. *WHA60.23 (2007) requests the development of a set of recommendations on marketing of foods and non-alcoholic beverages (FNAB) to children. In 2009, WHO initiates to aid Member states to develop nutrient profile models.*

c. *WHA 63.14 (May 2010) - WHO Member States endorses "A set of recommendations on the marketing of FNAB to children (as per WHA60.23, 2007).*

d. *Companies participated in Malaysia Pledges include Mondelez International (Kraft Foods as former name), F&N Dairies, F&N Manufacturing Sdn. Bhd, MARS, Nestle, Unilever, PepsiCo, Coca-Cola, Guan Chong Cocoa, Ace Canning, Kellogg's and Delfi Marketing as of September 2016.*

[START RECORDING, if skip Session A]

Session B Semi-structure questions related to restriction on unhealthy food promotion to children

[Content will be similar to Session A (i), but subject issue will be related to restriction on unhealthy food promotion to children.]

1. What were you doing at the time of the policy development and implementation?
   a. Can you share with me, how you have been involved in the development and/ or implementation of restriction on unhealthy food promotion to children?

   (Prompts – for secondary data: If you have not been directly involved, can you tell me what you know about the development and implementation of unhealthy food promotion to children)

2. **Development of Guideline for Fast Food Advertisements on TV 2007/ 08 and introduction of self-regulation approach via the Malaysian Pledge in 2013 were voluntary approaches to restrict unhealthy food promotion to children in Malaysia.**

   Prompts:

   a. What can you tell me about the process?
   (*Probe – was it smooth? or convoluted?*)

   b. Are you aware of factors contributed to this?
      i. Who was involved?
      (Probe – Government, industry, NGOs and etc. The role of National Food Safety and Nutrition Council (NFSNC) in 2012?)
      ii. What were the key arguments and support for developing the policy at that time?
      iii. Any key event which might have precipitated it to happen?
      iv. Can you relate if and how the media reported this issue?
      (Probe - What was the media coverage?)

c. Can you describe the scope of these policies when they were first proposed in the early development phase?
(Probe - It appears that the final policy was different from what was first proposed. Can you describe the factors / reasons why? What were the negotiations that went on to get to the final policy? E.g. scopes from legislative to self-regulatory).

[Instruction: Towards the end of the questions above, if the interviewee does not mention about the components and key prompts based on literature reviews, the following questions can be used as a guide to probe more specific information.]

d. [OPTIONAL AND EXAMPLE]

Keeping track on what we have been discussing so far, you have not mentioned on (use prompts e.g. government stakeholders outside Ministry of Health), which was indicated by other people or literature. Would you like to comment on this?

(If the interviewee indicates the key prompts, then you can probe further. For example – What were the external factors? When were these happened? How did they contribute to the policies development?)

*Prompts (*these components can be vice-versa):*

**Components**	**Key Prompts (do not mention, unless the interviewee indicates)**	**Probe, if necessary (do not mention, unless the interviewee indicates)**
Government	Coalition/ Partnership	Who, what, when, why and how
Private Sector	Leadership	Who, when, why and how
Civil Society	Public consultation	When, what and how
Facilitators	Political will/ awareness	When, how and why
Barriers	Cost	What, why or how
	Resources	What, why and how
	Internal and external factors	How, what and when

i. **Facilitators – leadership, positive response from public consultation.*
ii. *Government coalition – Ministry of Health and which other Ministries; established Expert Committee on Nutrition, Health Claims & Advertisement – involved nutritionists, dietitians, medical doctors, food scientists, academia.*
iii. *External factors e.g. media, advocacy from civil society, political events, economic conditions, influence from WHO, advocacy of international agenda such as the Global Strategy of Diet, Physical Activity and Health.*
iv. *Roles of private sector – forming coalition within industries (e.g. Federation of Malaysian Manufacturers), share common beliefs or vice-versa, applied resources e.g. media to advocate their agendas; corporate political activities highlighted by the food companies.*
v. **Barriers – low political awareness, incurred additional printing cost, lack of expertise and public advocacy.*

3. **From policies development, I am moving to the period after the introduction of Guideline for Fast Food Advertisement 2007/08 and Malaysian Pledge 2013. I would also like you to think about the future plan of the government, which is to introduce regulatory approach to restrict unhealthy food promotion to children.**

a. Based on your experience or observation, what can you tell me about the implementation of these policies?
   (Probe – was it smooth? or full of obstacles, convoluted? poorly, moderately or well implemented?)

b. Are you aware of factors that contributed to the implementation?

   Prompts, if necessary:

   i. Who was involved?
      (Probe – Government, industry, NGOs and etc.)
   ii. What were the key arguments and support for the implementation?
   iii. Any key event which might precipitate the implementation?
   iv. Did you observe any influence around policy content?
   v. Could you recall if there was any discussion in the media?
      (*Prompt also the future plan by the government)*

[Instruction: Towards the end of the questions above, if the interviewee does not mention about the components and key prompts based on literature reviews, the following questions can be used as a guide to probe more specific information.]

c. **[OPTIONAL AND EXAMPLE]**

It was a fascinating discussion and we covered some of the factors such as (recap what have been discussed). However, other people or literature highlighted (use prompts e.g. the roles of private sector), which you have not mentioned. Would you like to comment on this?

(If the interviewee indicates the key prompts, then you can probe further. For example – What are the resources? How did they contribute to the policies implementation?)

*Prompts (*these components can be vice-versa):*

Components	Key Prompts (do not mention, unless the interviewee indicates)	Probe, if necessary (do not mention, unless the interviewee indicates)
Government	Implementation approach	What, why, how
Private Sector	Organisation structure	What, who and how
Civil Society	Political will, perception and commitment	What and how
Facilitators	Resources	What, when and how
Barriers	A demand for change	What and why
	Internal and external factors	What, why and how

i. **Facilitators – proper collaborative approach; prioritised by the organisation (e.g. key performance index), structured framework (e.g. guideline, support) and trained staffs, external factors.*
ii. **Barriers – lack of individualised approach (e.g. top-down) and engagement with other departments, implementer's perception, organisation norm, high cost or profit margin reduction (e.g. loss of profit and employment, affect supply and demand paradigm), nature of organisation, difficult to integrate (e.g. vary format or parties involved which led to challenges and time consuming), low consumer demand, lack of evidence to support the formulation, difficulty in interpretation, lack of infrastructure* (e.g. human resources, no specialisation), existing legal or policy conflicts (e.g. self-regulation is stated in regulation), corporate political activities.*

4. If you could change the policy, what would you want to suggest?

Prompts: Who should do what and how? How do you think your ideas will make a difference to improve this policy?

Thank you very much for all the valuable information you shared with me.

We are almost done.

Session C: Corporate Political Activities

[Note: Food industry representatives will not be required to answer this session]

Lastly, we are interested to hear about the role or influence that the food industry may have in food policy development and implementation.

1. Can you share with me if you have observed/ experienced any involvement of the food industry in influencing policy outcomes related to [nutrition labelling and/or food marketing]?

   Prompt:

   i. What kind of activities have you observed?

   ii. What do you think was the impact of these strategies or practices?

2. **Beyond what we have been discussed related to nutrition labelling and food promotion**, have you observed any involvement of food industries in government food policy?

   Prompt:

   a. Can you tell me how this involvement could have impacted the government food policies?

[Instruction: Towards the end of the questions above, if the interviewee does not mention about the key prompts identified based on literature reviews, the following questions can be used as a guide to probe more specific information.]

b. [OPTIONAL AND EXAMPLE]

You mentioned about strategies such as (recap what have been discussed). Now, I am going to ask you specific questions related to the involvement of food industry in food policy development and implementation. It is completely fine if you choose not to answer. I will just note down your response accordingly.

As per the literature, we found (identify key prompts that are yet to be discussed e.g. donation and sponsorship) might be another practice which you have not mentioned. Would you like to comment on this?

(Probe, if necessary – Did you observe any of this? Can you tell me how this involvement could have impacted the government food policies?)

Key prompts:

**Practice**	**Examples**<br>**(Do not mention, unless the interviewee indicates)**
Lobbying	stress economic importance, frame the debate, shape the evidence and etc.
Donation or sponsorship	political parties or decision makers, NGOs, institutes
Seek for public supports	partnership with key leaders, NGOs, community, media and etc.
Prefer voluntary approach instead of regulations	Proposal of self-regulatory instead of legislation
Legal challenge	Use legal action or clauses in trade agreement or contract
Criticism	Prevent or counteract public health advocates

Ending Questions

1. How do you see the importance of monitoring and accountability of policy activities in improving this policy? *Describe your answer.*

2. Do you have or would you like to recommend us any material/ document (e.g. archives, journal, newspaper, press statement, guideline – A Guide for nutrition labelling and claims etc.) to refer for a broader overview on the policies discussed?

   a. *Before we finish, do you have anyone to recommend for an interview on policies and issues discussed? If yes, please write his/her name/contact on this piece of paper and put it in an envelope.*

Thank you for your precious time.
We are grateful that you have contributed
your valuable intellectual opinions in this Interview.

-- THE END ---
