# Supplementary material for: Tracking Progress from Policy Development to Implementation: A Case Study on Adoption of Mandatory Regulation for Nutrition Labelling in Malaysia
Source: Nutrients. 2021 Jan 29;13(2):457. doi: 10.3390/nu13020457 (PMC7910920; doi:10.3390/nu13020457)
Supplement: Supplementary file 1 [file nutrients-13-00457-s001.zip › 4. Supplementary Material 2 Participant profiles.pdf]

Supplementary Material 2 Participant Profiles

Background	Overall field of expertise	Education level (number of participants)	Year of experience (Mean±SD)	Number of participants contributed in the following section	
				Mandatory nutrition labelling case	CPA
Government	Food regulations, Codex, policy, standard or strategy development, programme planning and implementation, public health, prevention and control of non-communicable diseases, nutrition label, front-of-pack labelling, nutrition, dietetics, and international collaboration or engagement for nutrition and related strategies.	Degree (n=2)<br>Master (n=4)<br>PhD (nil)	21.0±10.1	6	6
Food industry	Nutrition, dietetics, food regulatory affairs (including food regulations compliance and technical meetings involvement in industry association and other stakeholders), and product development (i.e., leads R&D team).	Degree (n=2)<br>Master (n=1)<br>PhD (nil)	20.7±9.0	3	N/A
Civil society	Public health nutrition, nutrients in foods, food regulations, community nutrition, and consumer behaviours.	Degree (nil)<br>Master (nil)<br>PhD (n=3)	37.0±6.1	3	3
Overall		Degree (n=4)<br>Master (n=5)<br>PhD (n=3)	24.9±11.0	12	9

Abbreviations: CPA = Corporate Political Activity; N/A = Not applicable; nil = zero; PhD = Doctor of Philosophy; R&D = Research and development; SD = Standard deviation

Notes:

1. Food industry participants did not contribute in questions related to corporate political activities during the semi-structure interviews.

2. Participants might have more than one background. The research team assigned the most relevant background corresponding to their roles during the policy processes.
